# Polymorphisms in *PARP1* predict disease-free survival of triple-negative breast cancer patients treated with anthracycline/taxane based adjuvant chemotherapy

**DOI:** 10.1038/s41598-020-64473-8

**Published:** 2020-04-30

**Authors:** Yuqian Liao, Yulu Liao, Jun Li, Jianping Xiong, Ying Fan

**Affiliations:** 1 Department of Oncology, The First Affiliated Hospital of Nanchang University, Nanchang, Jiangxi Province China; 2 Department of Radiation Oncology, Jiangxi Cancer Hospital, Nanchang, Jiangxi province China; 3 Department of Medical Oncology, Cancer institute and hospital, Peking Union Medical college, Chinese Academy of Medical science, Beijing, P.R. China

**Keywords:** Cancer therapy, Chemotherapy

## Abstract

Triple-negative breast cancer (TNBC) is a highly aggressive disease and of poor prognosis. It is very important to identify novel biomarkers to predict therapeutic response and outcome of TNBC. We investigated the association between polymorphisms in *PARP1* gene and clinicopathological characteristics or survival of 272 patients with stage I-III primary TNBC treated with anthracycline/taxane based adjuvant chemotherapy. We found that after adjusted by age, grade, tumor size, lymph node status and vascular invasion, rs7531668 TA genotype carriers had significantly better DFS rate than TT genotype carriers, the 5 y DFS was 79.3% and 69.2% (*P* = 0.046, HR 0.526 95% CI 0.280–0.990). In lymph node negative subgroup, DFS of rs6664761 CC genotype carriers was much better than TT genotype carriers (*P* = 0.016, HR 0.261 95% CI 0.088–0.778) and DFS of rs7531668 AA genotype carriers was shorter than TT genotype carriers (*P* = 0.015, HR 3.361 95% CI 1.259–8.969). In subgroup of age ≤ 50, rs6664761 TC genotype predicted favorable DFS than TT genotype (*P* = 0.042, HR 0.405 95% CI 0.170–0.967). Polymorphisms in *PARP1* gene had no influence on treatment toxicities. After multivariate analysis, tumor size (*P* = 0.037, HR = 2.829, 95% CI: 1.063–7.525) and lymph node status (*P* < 0.001, HR = 9.943, 95% CI: 2.974–33.243) were demonstrated to be independent prognostic factors. Our results suggested that polymorphisms in *PARP1* gene might predict the DFS of TNBC patients treated with anthracycline/taxane based adjuvant chemotherapy.

## Introduction

Breast cancer is one of the most common cancers and is the leading cause of cancer-related death in women around the world^1^. According to molecular profile, breast cancer was divided into several intrinsic subtypes: luminal-A, luminal-B, HER2-enriched, basal-like, and a normal breast-like group^2^. Triple negative breast cancer (TNBC) is defined as lacking expression of estrogen receptor (ER), progesterone receptor (PR) and epidermal growth factor receptor 2 (HER2). It accounts for 15–20% of all breast cancers and is characterized by enhanced aggressiveness, young age of onset and poor prognosis^3^. All intrinsic subtypes can be found in TNBC, but 50%-75% of TNBC have basal phenotype^4^. Recently, four to six distinct subtypes have been defined within TNBC, such as basal-like and claudin-low^5^. Chemotherapy remains the main treatment for TNBC, but the overall survival for metastatic TNBC is only 13–18 months^6^. Though PARP inhibitors showed promising effect in BRCA mutation patients, their effectiveness in TNBC need to be further verified. So it is very important for us to explore novel biomarkers and potential therapeutic targets in TNBC patients^7^.

DNA damage caused by exogenous and endogenous factors plays an important role in carcinogenesis^8^. Multiple DNA repair pathways are vital for controlling DNA damage and maintaining genomic stability, such as base excision repair (BER) pathway^9^. Impaired DNA repair impacts upon carcinogenesis and response to DNA damaging radiotherapy and chemotherapeutics^10^. Poly[ADP-ribose] polymerase (PARP) is an abundant, highly conserved, cell signaling protein. The activation of PARP is essential for DNA single strand break (SSB) repair^11^, a sub-pathway related to BER. PARP-1, also known as ADPRT, is a major member of the PARP family, which is mainly responsible for the recognition of damaged bases and the recruitment of repaired proteins^12^.

It has been reported that PARP1 expression was correlated to clinicopathological variables and outcome of breast cancer patients^13^. Some investigators found that nuclear expression of PARP1 in invasive primary breast tumors is associated with chemotherapy sensitivity^14^. However, there is no systemic research about polymorphisms in *PARP1* and prognosis of TNBC patients. In our study, we first demonstrated that polymorphisms in *PARP1* gene were associated with survival of TNBC patients treated with anthracycline/taxane based adjuvant chemotherapy.

## Results

### Clinical characteristics and survival of TNBC patients

A cohort of 272 TNBC patients was enrolled in this study. The median age at diagnosis is 47 years old (range: 23–75). 166 (61.0%) patients were ≤ 50 years old. 203 (74.6%) patients were diagnosed with grade 3 tumors. Most patients were at stage II and III (189 patients, 69.5%). The 5-year OS and DFS rate were 86.9% and 72.2%, respectively. The survival rates for patients with different clinicopathological characteristics were listed in Table 1. Patients older than 50 years old had a significantly better 5 y DFS rate than those younger than 50 years old (79.4% vs. 68.0%, *P* = 0.044, HR = 0.572, 95%CI: 0.332–0.986). The Kaplan-Meier analysis revealed a significant higher DFS and OS for patients with tumor size ≤ 2 cm and negative lymph node metastasis. There was no significant association between grade, vascular invasion and TNBC survival. After multivariate analysis, tumor size (*P* = 0.037, HR = 2.829, 95%CI: 1.063–7.525) and lymph node status (*P* < 0.001, HR = 9.943, 95%CI: 2.974–33.243) were proved to be independent prognostic factors.

**Table 1:** Clinicopathological characteristics and survival of TNBC.

Variables	Patients (%)	5yDFS(%)	HR(95%CI)	*P*	5yOS(%)	HR(95%CI)	*P*
**Age**							
≤50	166(61.0)	68.0	1(Ref)		86.9	1(Ref)	
> 50	106(39.0)	79.4	0.572(0.332–0.986)	0.044	86.8	0.877(0.404–1.906)	0.740
**Grade**							
1–2	69(25.4)	68.1	1(Ref)		96.8	1(Ref)	
3	203(74.6)	73.5	1.070(0.608–1.882)	0.814	83.6	3.062(0.923–10.156)	0.067
**Vascular invasion**							
negative	254(93.4)	72.9	1(Ref)		87.0	1(Ref)	
positive	18(6.6)	62.2	1.529(0.659–3.547)	0.322	81.6	2.207(0.762–6.389)	0.144
**Tumor size**							
≤2 cm	126(46.3)	82.6	1(Ref)		95.5	1(Ref)	
>2 cm	146(53.7)	63.7	2.233(1.308–3.813)	0.003	80.2	3.953(1.502–10.400)	0.005
**Lymph node**							
negative	160(58.8)	80.0	1(Ref)		98.0	1(Ref)	
positive	112(41.2)	61.2	2.824(1.705–4.677)	<0.001	72.3	12.718(3.839–42.131)	<0.001
**TNM**							
I	83(30.5)	83.9	1(Ref)		100.0	1(Ref)	
II	136(50.0)	75.1	1.617(0.782–3.343)	0.195	92.8	5.738(0.734–44.851)	0.096
III	53(19.5)	46.8	6.130(2.968–12.663)	<0.001	53.5	34.099(4.528–256.803)	0.001

Abbreviations: DFS, disease free survival; OS, overall survival; HR, hazard ratios; CI, confidence interval; Ref, reference.

### Polymorphisms in *PARP1* and survival of TNBC patients

**Figure 1:**
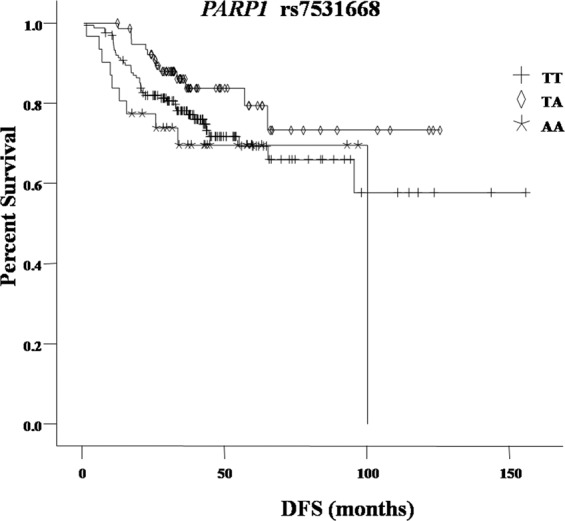
Kaplan-Meier curve of DFS for patients with different *PARP1* rs7531668 genotypes.

Tables 2, 3 listed the 5-year DFS and OS rate for patients with different genotypes. After adjusted by age, grade, tumor size, lymph node status and vascular invasion, rs7531668 TA genotype carriers had significantly better DFS rate than TT genotype carriers, the 5 y DFS was 79.3% and 69.2% (*P* = 0.046, HR 0.526 95% CI 0.280–0.990) (Fig. 1), respectively. There was no association between other polymorphisms in *PARP1* and survival of TNBC patients.

**Table 2:** *PARP1* genotypes and disease-free survival.

Variables	Patients (%)	5 y DFS(%)	Crude		Adjusted	
			HR (95% CI)	*P*	HR (95% CI)	*P*
**Rs1136410**						
TT	94(34.6)	69.4	1(Ref)		1(Ref)	
TC	134(49.3)	73.3	0.791(0.463–1.351)	0.391	0.891 (0.516–1.537)	0.678
CC	44(16.1)	74.2	0.895(0.440–1.819)	0.759	0.877(0.428–1.796)	0.719
**rs11801168**						
TT	7(2.6)	64.3	1(Ref)		1(Ref)	
TC	68(25.0)	76.7	0.885(0.202–3.881)	0.872	1.070(0.240–4.772)	0.929
CC	197(72.4)	71.4	0.996(0.241–4.109)	0.995	1.255(0.296–5.322)	0.758
**rs12568297**						
GG	226(83.1)	72.4	1(Ref)		1(Ref)	
GC	41(15.1)	75.1	1.085(0.552–2.133)	0.814	1.020(0.513–2.027)	0.956
CC	5(1.8)	53.3	1.511(0.366–6.229)	0.568	1.531(0.351–6.680)	0.571
**Rs6664761**						
TT	33(12.1)	68.3	1(Ref)		1(Ref)	
TC	119(43.8)	74.6	0.537(0.261–1.102)	0.090	0.564(0.272–1.171)	0.124
CC	120(44.1)	70.5	0.712(0.358–1.417)	0.333	0.755(0.376–1.516)	0.429
**Rs7531668**						
TT	163(59.9)	69.2	1(Ref)		1(Ref)	
TA	78(28.7)	79.3	0.594(0.319–1.108)	0.102	0.526(0.280–0.990)	0.046
AA	31(11.4)	69.6	1.335(0.669–2.662)	0.412	1.190(0.588–2.406)	0.629

Abbreviations: DFS, disease free survival; OS, overall survival; HR, hazard ratios; CI, confidence interval; Ref, reference.

**Table 3:** *PARP1* genotypes and overall survival.

Variables	5 y OS(%)	Crude		Adjusted	
		HR (95% CI)	*P*	HR (95% CI)	*P*
**rs1136410**					
TT	87.3	1(Ref)		1(Ref)	
TC	90.5	0.578(0.239–1.395)	0.222	0.818(0.334–2.008)	0.662
CC	77.1	1.382(0.555–3.439)	0.487	1.572(0.607–4.073)	0.352
**rs11801168**					
TT	85.7	1(Ref)		1(Ref)	
TC	91.5	0.344(0.066–1.788)	0.204	0.663 (0.123–3.570)	0.632
CC	85.3	0.486(0.113–2.090)	0.332	0.990(0.214–4.577)	0.990
**Rs12568297**					
GG	85.5	1(Ref)		1(Ref)	
GC	95.0	0.470(0.111–1.989)	0.305	0.451(0.105–1.942)	0.285
CC	80.0	2.823(0.658–12.110)	0.162	1.678(0.343–8.214)	0.523
**Rs6664761**					
TT	85.2	1(Ref)		1(Ref)	
TC	90.4	0.772(0.205–2.915)	0.703	1.079(0.279–4.178)	0.912
CC	84.4	1.522(0.446–5.196)	0.503	1.974(0.563–6.919)	0.288
**Rs7531668**					
TT	84.3	1(Ref)		1(Ref)	
TA	91.4	0.486 (0.183–1.289)	0.147	0.483(0.180–1.292)	0.147
AA	90.4	0.499(0.117–2.130)	0.348	0.376(0.087–1.628)	0.191

Abbreviations: DFS, disease free survival; OS, overall survival; HR, hazard ratios; CI, confidence interval; Ref, reference.

### **Polymorphisms in*PARP1*gene and survival of TNBC in different subgroups**

Since there were only 7 and 5 patients with rs11801168 TT and rs12568287 CC genotypes, we only performed subgroup analysis for rs1136410, rs6664761 and rs7531668 (Table 4). In lymph node negative subgroup, DFS of rs6664761 CC genotype carriers was much better than TT genotype carriers (*P* = 0.016, HR 0.261 95% CI 0.088–0.778) and DFS of rs7531668 AA genotype carriers was shorter than TT genotype carriers (*P* = 0.015, HR 3.361 95% CI 1.259–8.969). In subgroup of age ≤50, rs6664761 TC genotype predicted favorable DFS than TT genotype (*P* = 0.042, HR 0.405 95% CI 0.170–0.967). No significant relationship was observed between polymorphisms and OS in any subgroups.

**Table 4:** Subgroup analysis of polymorphisms and survival.

subgroup	variants	DFS	
		HR(95%CI)	*P*
Lymph node negative	rs1136410		
	TT	1(Ref)	
	TC	0.937(0.404–2.170)	0.879
	CC	0.219(0.027–1.716)	0.148
	Rs6664761		
	TT	1(Ref)	
	TC	0.435(0.161–1.180)	0.102
	CC	0.261(0.088–0.778)	0.016
	Rs7531668		
	TT	1(Ref)	
	TA	1.083(0.406–2.890)	0.873
	AA	3.361(1.259–8.969)	0.015
Age ≤ 50	rs1136410		
	TT	1(Ref)	
	TC	0.683(0.353–1.319)	0.256
	CC	1.073(0.498–2.315)	0.857
	Rs6664761		
	TT	1(Ref)	
	TC	0.405(0.170–0.967)	0.042
	CC	0.697(0.314–1.545)	0.374
	Rs7531668		
	TT	1(Ref)	
	TA	0.530(0.243–1.154)	0.110
	AA	1.711(0.785–3.731)	0.177

Abbreviations: DFS, disease free survival; HR, hazard ratios; CI, confidence interval; Ref, reference; UK, unknown.

### **Polymorphisms in*PARP1*gene and toxicities induced by anthracycline/taxane based chemotherapy**

We further investigated the relationship between different genotypes and the risk of hematological (neutropenia, thrombocytopenia and anemia) and non-hematological toxicities (nausea, vomiting and neuropathy). We analyzed the distribution of genotypes between patients with or without chemotherapy induced toxicities. The results indicated that polymorphisms in *PARP1* had no influence on either hematological or non-hematological toxicities (Tables 5, 6).

**Table 5:** *PARP1* genotypes and hematological toxicities.

Variables	Neutropenia		Thrombocytopenia		Anemia	
**Rs1136410**	*P*	HR (95%CI)	*P*	HR (95%CI)	*P*	HR (95%CI)
TT		1(Ref)		1(Ref)		1(Ref)
TC	0.635	0.880 (0.519–1.492)	0.212	0.521 (0.187–1.451)	0.832	0.895 (0.321–2.494)
CC	0.550	0.803 (0.392–1.646)	0.477	1.491 (0.496–4.487)	0.067	2.762 (0.932–8.183)
**rs11801168**						
TT		1(Ref)		1(Ref)		1(Ref)
TC	0.991	1.009 (0.209–4.859)	0.845	0.800 (0.085–7.529)	0.640	0.581 (0.060–5.659)
CC	0.644	0.699 (0.152–3.203)	0.443	0.424 (0.047–3.789)	0.609	0.567 (0.064–4.986)
**Rs12568297**						
GG		1(Ref)		1(Ref)		1(Ref)
GC	0.442	1.301 (0.666–2.541)	0.269	1.823 (0.629–5.286)	0.378	1.605 (0.560–4.596)
CC	0.646	1.527 (0.250–9.312)	0.301	3.281 (0.346–31.116)	0.354	2.889 (0.306–27.234)
**Rs6664761**						
TT		1(Ref)		1(Ref)		1(Ref)
TC	0.370	1.427 (0.655–3.108)	0.313	2.936 (0.362–23.808)	0.313	2.936 (0.362–23.808)
CC	0.307	1.500 (0.689–3.265)	0.270	3.229 (0.402–25.971)	0.199	3.888 (0.490–30.870)
**Rs7531668**						
TT		1(Ref)		1(Ref)		1(Ref)
TA	0.900	0.966 (0.563–1.658)	0.698	0.822 (0.306–2,208)	0.500	0.716 (0.271–1.893)
AA	0.299	0.663 (0.305–1.441)	0.290	0.329 (0.042–2.585)	0.233	0.286 (0.037–2.234)

Abbreviations: HR, hazard ratios; CI, confidence interval; Ref, reference.

**Table 6:** *PARP1* genotypes and non-hematological toxicities.

Variables	Nausea		Vomiting		Neuropathy	
**Rs1136410**	*P*	HR (95%CI)	*P*	HR (95%CI)	*P*	HR (95%CI)
TT		1(Ref)		1(Ref)		1(Ref)
TC	0.848	1.054 (0.614–1.811)	0.883	1.060 (0.484–2.321)	0.978	1.008 (0.573–1.774)
CC	0.296	1.471 (0.714–3.032)	0.303	0.500 (0.134–1.871)	0.779	0.895 (0.410–1.950)
**rs11801168**						
TT		1(Ref)		1(Ref)		1(Ref)
TC	0.634	0.681 (0.141–3.305)	0.845	0.800 (0.085–7.529)	0.779	1.278 (0.230–7.101)
CC	0.991	0.991 (0.216–4.547)	0.868	0.832 (0.096–7.215)	0.893	1.121 (0.212–5.941)
**Rs12568297**						
GG		1(Ref)		1(Ref)		1(Ref)
GC	0.057	0.488 (0.233–1.021)	0.324	0.536 (0.155–1.850)	0.985	0.993 (0.486–2.030)
CC	0.896	0.887 (0.145–5.409)	0.641	1.698 (0.183–15.726)	0.579	0.535 (0.059–4.870)
**Rs6664761**						
TT		1(Ref)		1(Ref)		1(Ref)
TC	0.481	0.756 (0.347–1.647)	0.900	0.933 (0.317–2.752)	0.438	1.402 (0.597–3.294)
CC	0.634	0.828 (0.381–1.799)	0.325	0.565 (0.182–1.760)	0.761	1.143 (0.484–2.700)
**Rs7531668**						
TT		1(Ref)		1(Ref)		1(Ref)
TA	0.439	0.802 (0.459–1.402)	0.979	0.989 (0.425–2.298)	0.606	1.163 (0.656–2.060)
AA	0.453	1.343 (0.622–2.902)	0.490	1.457 (0.500–4.249)	0.544	0.764 (0.320–1.823)

Abbreviations: HR, hazard ratios; CI, confidence interval; Ref, reference.

## Discussion

PARP1 is the major member of PARP family. It was identified by Chambon *et al*. in 1963 as a protein whose enzymatic activity allows it to generate ADP-ribose polymers^15^. DNA double-strand breaks (DSBs) can lead to fragmentation, loss or rearrangement of chromosomes^16^. DSBs are repaired through two pathways: homologous recombination (HR) and error-prone non-homologous end joining (NHEJ), which repair DSBs generated during the S-phase and outside the S-phase of the cell cycle respectively^17^. PARP1 is involved in both HR and NHEJ pathways^18,19^. In our study, we systemically investigated the association between polymorphisms in *PARP1* and prognosis of TNBC, we first demonstrated that rs7531668 was related to DFS of all patients and lymph node negative patients and rs6664761 genotype predicted DFS in lymph node negative and age ≤ 50 subgroups. We also found that polymorphisms in *PARP1* gene had no influence on treatment toxicities.

*PARP1* gene resides on the long arm of chromosome 1. It spans 23 exons and 22 introns^13^. Till now, there is no report about polymorphisms investigated in our study and prognosis of breast cancer. However, the expression level of PARP1 has been reported to be associated with the survival of breast cancer patients, but the results are inconsistent. Rojo *et al*. found that nuclear PARP-1 is overexpressed during the malignant transformation of the breast, particularly in triple-negative tumors, and independently predicts poor prognosis in operable invasive breast cancer^20^. Donizy *et al*. reported that nuclear-cytoplasm expression (NCE) of PARP-1 was associated with unfavorable prognosis in lymph node negative early breast cancer^21^. While in Aiad’s study, the authors demonstrated that PARP-1 immunohistochemical expression is a marker of good prognosis in locally advanced breast cancer^22^. A meta-analysis included 3506 patients from eight studies, the results indicated that higher PARP expression indicated a worse clinical outcome in early stage breast cancer, with a HR of 3.08 (95% CI, 1.14 ± 8.29, P = 0.03) for disease-free survival and a HR of 1.82 (95% CI, 1.20 ± 2.76; P = 0.005) for overall survival^23^. But in locally advanced breast cancer, the authors observed no association between PARP expression level and survival^23^. The results from above studies suggested that PARP1 protein might be a stronger prognostic marker in early stage patients. In our study, two polymorphisms were associated with DFS in lymph node negative patients but not in lymph node positive patients, which supported the results from above researches.

PARP1 was also related to sensitivity of some chemotherapeutic agents. Minckwitz *et al*. found that high cPARP expression predicted high sensitivity to neoadjuvant taxane/anthracycline-based chemotherapy^24^. Zhai *et al*. found that higher nuclear PARP1 expression correlated with increased *in vitro* chemosensitivity against docetaxel and epirubicin but not cisplatin and vinorelbine^14^. In their study, patients with high nPARP1 expression were more sensitive to anthracycline/taxane based neoadjuvant chemotherapy and with higher pathologic responses^14^. Results from Egyptian researchers also demonstrated that PARP1 immunohistochemical expression is predictive of response to anthracycline/taxane based neoadjuvant chemotherapy in locally advanced breast cancer patients^22^. As PARP1 involves in repairing DNA double-strand breaks, it is easy for us to understand its influence on sensitivity of agents which cause DNA damage, such as anthracyclines. While PARP1 may also lead to an intrinsic cell death program (PARP1-dependent cell death)^25^, which might explain its effect on the sensitivity of other drugs, such as taxanes.

Polymorphisms in *PARP1* have been reported to be associated with the risk of several kinds of cancers. In one meta-analysis, the authors found that rs1136410 may be involved in cancer development at least in some ethnic groups (Asian) or some specific cancer types (gastric, cervical, and lung cancers, and glioma)^26^. Alanazi *et al*. confirmed that rs1136410 was associated with risk of breast cancer in Saudi population^27^. Rs1136410 is the mostly investigated polymorphism in *PARP1* which locates at codon 762 in exon 17. Rs1136410 leads to a valineto-alanine substitution in the catalytic domain and then reduces the activities of PARP1^28^. *In vitro* enzymatic analysis of PARP1-Ala762 and PARP1-Val762 demonstrated that PARP1-Ala762 displayed 57.2% of the activity of PARP1-Val762 for auto-poly(ADP-ribosyl)ation and 61.9% of the activity of PARP1-Val762 for trans-poly(ADP-ribosyl)ation of histone H1^28^. As expression level of PARP1 protein was proved to predict prognosis and chemotherapy sensitivity in breast cancer patients^20,21^, we assumed that rs1136410 genotypes might relate to prognosis of TNBC. But in our study, no significant association was observed. There are some reasons for this result. Firstly, the prognostic value might vary between different ethnic groups. Secondly, the complicated interactions with other polymorphisms could also affect the results. Thirdly, all patients in our study received taxane/anthracycline-based adjuvant chemotherapy, which might compromise the prognostic value of PARP1, since higher PARP1 expression level has been found to predict higher sensitivity of taxanes and anthracyclines^24^. The underlying mechanisms of other SNPs on survival of TNBC are not yet clear and are going to be investigated in our further studies.

In conclusion, our results first demonstrated that polymorphisms in *PARP1* were associated with survival of TNBC patients receiving anthracycline/taxane based adjuvant chemotherapy especially in lymph node negative and age ≤ 50 subgroups. This study supported the findings from previous researches of PARP1 protein. We found no association between these polymorphisms and toxicities induced by chemotherapy. Since other polymorphisms have never been reported except rs1136410, further investigations are needed to verify the results.

## Materials and methods

### Patients

In our study, a total of 272 patients with stage I-III primary TNBC treated with anthracycline/taxane based adjuvant chemotherapy were enrolled between January 2004 and December 2014. Stage was determined according to American Join Committee on Cancer 2010 classification^29^. TNBC was defined according to guidelines issued by the American Society of Clinical Oncology (ASCO) and the College of American Pathologists (CAP) in 2010^30,31^. This investigation was approved by the Institutional Review Board of the Chinese Academy of Medical Sciences Cancer Hospital and Jiangxi Cancer Hospital. It was conducted in accordance with the ethical standards of the Declaration of Helsinki and following the national and international guidelines. All patients have consented to their blood and clinical information being used in this study. Clinical and pathological data were collected. Patients were followed until December 2018 to collect data on recurrence and death.

### Single nucleotide polymorphism selection and genotyping

Genotype data from *PARP1* gene regions encompassing 10 kb of upstream and 10 kb of downstream flanking sequences were extracted from the HapMap We used Chinese Han population. Haploview 4.2 software was to identify Tag SNPs. The inclusion criteria were: 1) SNPs known in ethnic Han Chinese population; 2) a minor allele frequency (MAF) > 0.05 and r^2^ > 0.8. A total of 5 candidate SNPs were selected for genotyping (Table 7).

**Table 7:** Information for the SNPs genotyped in this study.

SNPs	Position	Location	Alleles	MAF
rs1136410	1:226367601	coding sequence variant	C/T	0.1969
rs11801168	1:226357130	downstream transcript variant	T/C	0.4036
rs12568297	1:226356905	downstream transcript variant	G/C	0.2400
rs6664761	1:226362490	intron variant	T/C	0.2392
rs7531668	1:226408318	upstream transcript variant	T/A	0.2632

Abbreviations: SNP, single nucleotide polymorphism; MAF, minor allele frequency.

Genomic DNA was extracted from the peripheral blood samples of each patient and was isolated by the routine phenol–chloroform method. Primers and probes were designed by MassARRAY Typer 4.0 software. MassARRAY MALDI-TOF System (Sequenom Inc., San Diego, CA, USA)^32,33^ was used for genotyping by the method described in the Sequenom Genotyping Protocol.

### Statistical analyses

SPSS 18.0 statistical software (SPSS Inc, Chicago, IL, USA) was used for analysis. 5-year DFS and OS rates of patients with different genotypes were estimated by Kaplan–Meier product limit method and compared by the log-rank test. Hazard ratios of recurrence/metastasis and death with 95% confidence intervals (CI) were estimated by Cox regression model. The multivariate analysis was adjusted for age, histological grade, tumor size, lymph node status and vascular invasion. The distribution of genotypes in patients with or without toxicities were compared by two-sided Pearson’s Chi-square tests, odds ratios (ORs) and 95% confidence intervals (CI) were calculated by logistic regression. All statistical tests were two-sided, and P < 0.05 was considered significant.

### Ethical Standards

This article does not contain any studies with human or animal subjects performed by any of the authors.
